# Anticancer Phenolics from *Dryopteris fragrans* (L.) Schott

**DOI:** 10.3390/molecules23030680

**Published:** 2018-03-17

**Authors:** Zhen-Dong Liu, Dan-Dan Zhao, Shuai Jiang, Bei Xue, Yan-Long Zhang, Xiu-Feng Yan

**Affiliations:** 1 Key Laboratory of Saline-alkali Vegetation Ecology Restoration, Ministry of Education/Alkali Soil Natural Environmental Science Center, Northeast Forestry University, Harbin 150040, China; liu304418091@126.com; 2 Sino-Russian Joint Laboratory of Bioactive Substance, College of Life Science, Heilongjiang University, Harbin 150080, China; zhaodandan@hlju.edu.cn (D.-D.Z.); jiangshuaixbxb@foxmail.com (S.J.); 3 Department of Food Science, Tibet Agriculture and Animal Husbandry University, Tibet 860000, China; 13658940092@126.com

**Keywords:** *Dryopteris fragrans* (L.) Schott, Fragranoside B, anticancer activity

## Abstract

Cancer is one of the most major diseases that threatens human health and life. The aim of this work was to obtain novel anticancer molecules from *D. fragrans*, a kind of medicinal plant. The structure of the new compound was identified using spectroscopic data (^1^H-NMR, ^13^C-NMR and two dimensions NMR). Its anticancer properties were evaluated using the 3-(4,5-dimethyl-2-thiazolyl)-2,5-diphenyl-2-*H*-tetrazolium bromide (MTT) assay against four human cells including lung cancer cells (A549), breast cancer cells (MCF-7), gastric cancer cells (SGC7901) and noncancerous human umbilical vein endothelial cells (HUVEC). A new phenylpropanoid—(*E*)-caffeic acid-9-*O*-β-d-xylpyranosyl-(1→2)-β-d-glucopyranosyl ester (**1**), with seven known compounds (**2**–**8**)—was isolated. The IC_50_ value of compound **1** against MCF-7 cells was 2.65 ± 0.14 µM, and the IC_50_ values of compound **8** against three cancer cells were below 20 µM.

## 1. Introduction

**Figure 1:**
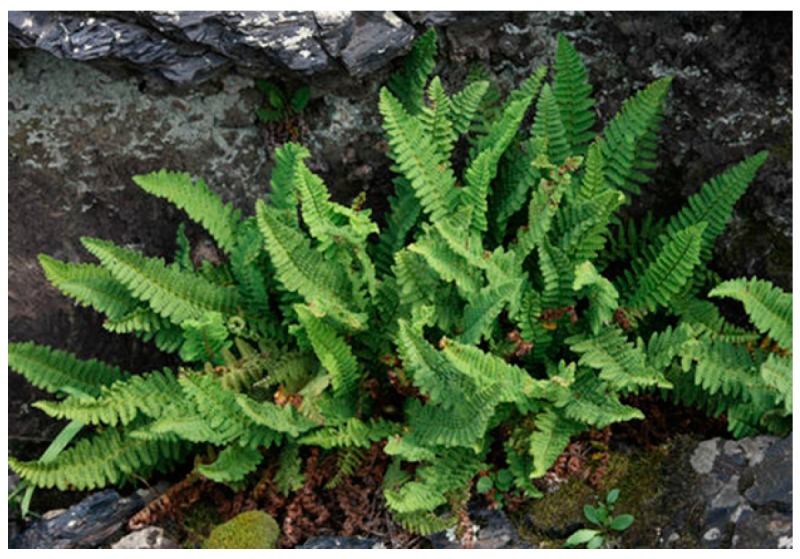
*D. fragrans* plant.

*D. fragrans* (Figure 1), from the *Dryopteris* genus of the Dryopteris family, is a deciduous perennial herb that is mostly distributed in Northeast China, Korea Japan, Russia and North America. [1,2]. The chemical components isolated from *D. fragrans* have exerted many biological effects [3,4,5,6]. In recent years, there have been many studies regarding the anticancer components of *D. fragrans*. Zhao [7] isolated a new coumarin—Dryofracoumarin A—from *D. fragrans*, which has cytotoxic activity. Su [8] reported that Dryofragin, a phloroglucinol derivative from *D. fragrans*, could stop human osteosarcoma U2OS cells from migrating and invading by lowering MMP-2 and MMP-9 expression and up-regulating the expression of TIMP-2 and TIMP-1 through the p38 MAPK and PI3K/AKT signal pathways. Zhong [9] obtained a novel sesquiterpene—Dryofraterpene A—from *D. fragrans*, which could significantly inhibit the proliferation of five kinds of cancer cell lines.

Thus, to discover new anticancer molecules, the chemical components of *D. fragrans* and its anticancer bioactivity were studied. This work can provide new resources for research and the development of new drugs against cancer.

## 2. Results and Discussion

### 2.1. Identification of Isolated Compounds

**Figure 2:**
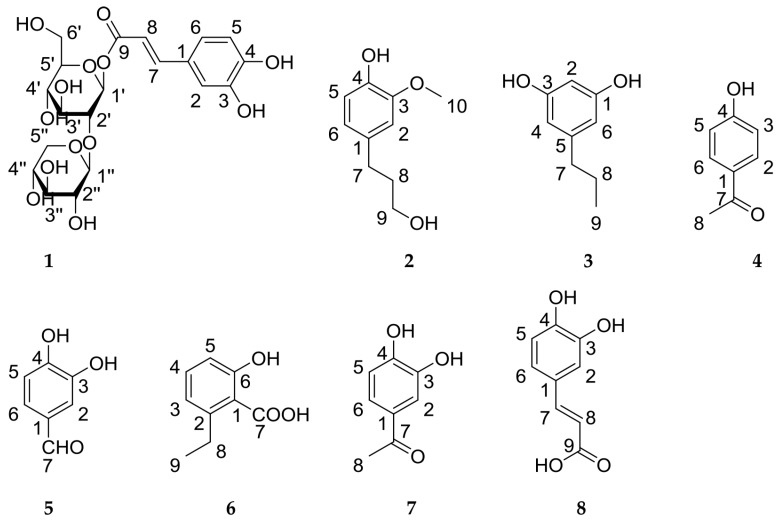
Structures of **1**–**8** isolated from *D. fragrans*.

Compound **1** (Figure 2) was puce powder. Its acid hydrolysis yielded d-glucose and d-xylose [10]. The molecular formula—C_20_H_26_O_13_—was deduced from High Resolution Electrospray Ionization Mass Spectrometry (HR-ESI-MS) with an [M + Na]^+^ peak at 497.1274 (calcd. for 497.1271). In the infrared spectrum, it displayed a hydroxyl group (3412 cm^−1^), a carbonyl group (1706 cm^−1^), a double-bond group (1680 cm^−1^) and a benzene ring group (1602 cm^−1^).

The ^1^H-NMR spectrum (Table 1) exhibited three proton signals at *δ* 7.05 (d, *J* = 2.0 Hz), *δ* 6.78 (d, *J* = 8.0 Hz) and *δ* 6.96 (dd, *J* = 2.0, 8.0 Hz), corresponding to a 1,3,4-trisubstituted phenyl group. Moreover, it showed two trans-double-bond protons at *δ* 7.64 (d, *J* = 15.8 Hz) and *δ* 6.26 (d, *J* = 15.8 Hz). There were two anomeric proton signals at *δ* 5.68 (d, *J* = 7.6 Hz) and *δ* 4.48 (d, *J* = 7.4 Hz), suggesting two sugars of β-type [11].

In the ^13^C-NMR spectrum and DEPT spectrum, it showed an ester carbonyl carbon (*δ* 167.3) and a pair of trans-double-bond carbons at *δ* 148.2 and *δ* 114.5.

**Figure 3:**
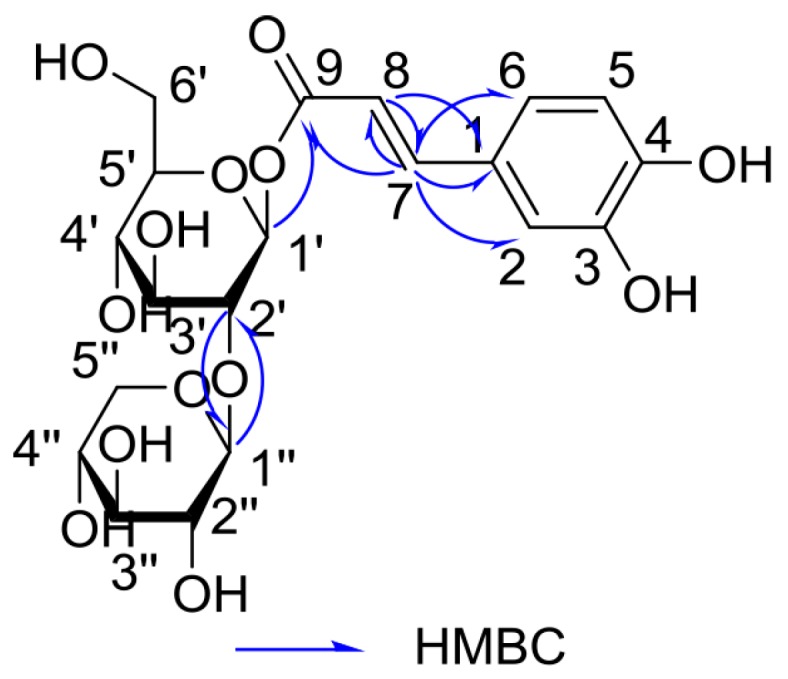
Heteronuclear Multiple Bond Correlation (HMBC) correlations of **1**.

In the ^1^H Detected Heteronuclear Multiple Bond Correlation (HMBC) spectrum (Figure 3), the correlation of the anomeric proton signal of β-d-glucose at *δ* 5.68 with the ester carbonyl carbon at *δ* 167.3 indicated that the ester carbonyl group was located at C-1′. The linkage between C-1′′ from β-D-xylose and C-2′ from β-d-glucose by oxygen was revealed through the HMBC correlations of H-1′′/C-2′. On the basis of nuclear magnetic resonance (NMR) data and the relevant literature [12], compound **1** was identified as (*E*)-caffeic acid-9-*O*-β-d-xylpyranosyl-(1→2)-β-d-glucopyranosyl ester. The new compound was trivially named Fragranoside B.

The known compounds (Figure 2) were identified as dihydroconiferyl alcohol (**2**) [13], 1,3-dihydroxyl-5-propylbenzene (**3**) [14], 4-hydroxyacetophenone (**4**) [15], 3,4-dihydroxybenzaldehyde (**5**) [16], 2-ethyl-6-hydroxybenzoic acid (**6**) [17], 3,4-dihydroxyacetophenone (**7**) [18] and caffeic acid (**8**) [19] through a comparison with the NMR data in the literature.

### 2.2. In Vitro Cytotoxicity Assay

A549, MCF-7, SGC7901 and human umbilical vein endothelial (HUVEC) cells were measured for cytotoxicity using the MTT assay with taxol as the positive control. As shown in Table 2, compound **1** showed significant inhibitory activity against MCF-7 cells with an IC_50_ value of 2.65 ± 0.14 μM. Compound **8** showed good cytotoxic activity against A549, MCF-7 and SGC7901 cells with IC_50_ values of 8.96–19.44 μM. However, the others were not active (IC_50_ > 50 μM). Notably, compound **1** exhibited no cytotoxic activities against A549, SGC7901 and noncancerous HUVEC cells. It showed good selectivity toward MCF-7. Thus, compound **1** can be considered as a promising lead compound and its anticancer mechanism should be further studied.

## 3. Materials and Methods

### 3.1. General Procedures

1D and 2D NMR spectra were obtained using a Bruker AM-400 (Bruker, Fällanden, Switzerland) instrument with tetramethyl silane as the internal standard. HR-ESI-MS was performed on VG Autospec-3000 mass spectrometers (VG, Manchester, UK). Semi-preparative High Performance Liquid Chromatography (HPLC) was performed using an Agilent 1100 liquid chromatography (Agilent Technologies, Waldbronn, Germany). Silica gel (200–300 mesh, Haiyang Chemical Co. Ltd., Qingdao, China) and Sephadex LH-20 (RuiDaHengHui Science and Technology Development Co., Ltd., Beijing, China) were used for column chromatography.

### 3.2. Plant Material

*D. fragrans* was collected from Wudalianchi City, Heilongjiang Province, China, in July 2015 and identified by Prof. Zhen-Yue Wang (Heilongjiang University of Chinese Medicine). The voucher specimen (No. XLMJ-20150828) of this plant was deposited in the Herbarium of Heilongjiang University of Chinese Medicine, Harbin, China.

### 3.3. Extraction and Isolation

The dried and powdered whole *D. fragrans* (8 kg) was extracted three times with water vapor. The concentrated extract (640 g) was fractionated by AB-8 macroporous resin eluted with EtOH–H_2_O (0:100, 30:70, 60:40, 95:5, *v*/*v*). The EtOH–H_2_O (30:70) extract (45 g) was chromatographed by silica gel eluted with CHCl_3_–MeOH (95:5, 90:10, 85:15, 80:20 and 50:50, *v*/*v*) to yield five fractions (A–E), respectively. Fraction A was subjected to column chromatography on silica gel eluted with CHCl_3_–MeOH (100:0, 98:2, 90:10 and 0:100, *v*/*v*) to afford four subfractions (A1–A4). Compound **2** (22 mg) was obtained from subfraction A1 by Sephadex LH-20 column chromatography with CHCl_3_. Compound **3** (3 mg) was purified by semi-preparative HPLC using MeOH–H_2_O (37:63, *v*/*v*) from subfraction A3. Fraction B was chromatographed by silica gel column (CHCl_3_–MeOH, 90:10, *v*/*v*), Sephadex LH-20 column (CHCl_3_) and semi-preparative HPLC (MeOH–H_2_O, 45:55, *v*/*v*) to give compound **4** (8 mg). Fraction C was separated by silica gel (CHCl_3_–MeOH, 80:20, *v*/*v*), Sephadex LH-20 column chromatography (CHCl_3_) and recrystallization to afford compound **5** (12 mg). Fraction D was repeatedly chromatographed using silica gel columns (CHCl_3_–MeOH) to yield three subfractions (D1–D3). Subfraction D1 was subject to Sephadex LH-20 columns chromatography (CHCl_3_) and recrystallization to yield compound **6** (20 mg). Compounds **7** (4 mg) and **8** (10 mg) were obtained from subfraction D3 by Sephadex LH-20 columns chromatography (CHCl_3_) and recrystallization. Compound **1** (9 mg) was isolated by column chromatography using Sephadex LH-20 (CHCl_3_–MeOH, 50:50, *v*/*v*), silica gel and semi-preparative HPLC (MeOH–H_2_O, 15:85, *v*/*v*) from fraction E.

### 3.4. Acid Hydrolysis

Compound **1** (5 mg) was hydrolyzed with 10 mL of 0.01 M H_2_SO_4_ for 4 h at 100 °C. After cooling, the hydrolysate was neutralized by 0.02 M KOH then extracted by CH_2_Cl_2_. The sugars in the aquatic layer were monitored by Thin-Layer Chromatography (TLC) with BuOH–H_2_O–AcOH (40:10:50, *v*/*v*/*v*, upper BuOH layer) as a developing system when compared with authentic sugars. The TLC plate was sprayed with a vanillin–H_2_SO_4_ solvent [20]. 

### 3.5. Cell Culture

Human A549, MCF-7, SGC7901 and HUVEC cells were obtained from the Cell Library of Committee on Type Culture Collection of Chinese Academy of Sciences (Shanghai, China). Cells were cultured at 37 °C, 5% CO_2_ in the Roswell Park Memorial Institute (RPMI) medium containing 10% FBS, 100 U/mL penicillin and 100 U/mL streptomycin.

### 3.6. MTT Assay

Anticancer activity was evaluated by the MTT assay. Compounds **1**–**8** were dissolved in dimethyl sulfoxide (DMSO) and diluted with RPMI medium for appropriate concentrations (0 μM, 0.08 μM, 0.4 μM, 2 μM, 10 μM and 50 μM). A549, MCF-7, SGC7901 and HUVEC cells were seeded in 96-well microtiter plates (100 μL, 5000 cells/well). After 24 h, the medium was removed and 100 μL of tested compounds with various concentrations were added into 96-well microtiter plates for 48 h. Next, 10 μL of MTT was added and the 96-well microtiter plates were incubated for another 4 h. The medium was removed and 150 μL DMSO was added to each well to dissolve the formazan crystals. The absorbance was measured by microplate spectrophotometer (Molecular Devices, Palo Alto, CA, USA) at 570 nm. Taxol was used as the positive control (0 μM, 0.01 μM, 0.04 μM, 0.16 μM, 0.64 μM, and 2.56 µM for 48 h). Half Maximal Inhibitory Concentration (IC_50_) values were calculated by GraphPad Prism. Data were obtained from three independent assays.

## 4. Conclusions

A new phenylpropanoid, (*E*)-caffeic acid-9-*O*-β-d-xylpyranosyl-(1→2)-β-d-glucopyranosyl ester (**1**), and seven known phenolics (**2**–**8**) were isolated from the medicinal plant *D. fragrans*. Compounds **1** and **8** showed good anticancer activity.

## Figures and Tables

**Table 1:** ^13^C-NMR (100 MHz) and ^1^H-NMR (400 MHz) spectral data of compound **1** in MeOD.

No.	*δ*_C_	*δ*_H_ (*J* in Hz)	No.	*δ*_C_	*δ*_H_ (*J* in Hz)
1	127.6 (C)		1′	94.3 (CH)	5.68 (d, 7.6)
2	115.1 (CH)	7.05 (d, 2.0)	2′	83.5 (CH)	3.60 (m)
3	146.9 (C)		3′	77.6 (CH)	3.65 (m)
4	149.9 (C)		4′	70.7 (CH)	3.42 (m)
5	116.5 (CH)	6.78 (d, 8.0)	5′	78.7 (CH)	3.40 (m)
6	123.2 (CH)	6.96 (dd, 8.0, 2.0)	6′	62.2 (CH_2_)	3.84 (dd, 1.6, 12.5), 3.68 (d, 6.3)
7	148.2 (CH)	7.64 (d, 15.8)	1′′	106.6 (CH)	4.48 (d, 7.4)
8	114.5 (CH)	6.26 (d, 15.8)	2′′	75.7 (CH)	3.18 (m)
9	167.3 (C)		3′′	77.5 (CH)	3.30 (m)
			4′′	71.0 (CH)	3.32 (m)
			5′′	67.4 (CH_2_)	3.70 (d, 5.3), 3.14 (m)

**Table 2:** Cytotoxicity of compounds **1**–**8** against A549, MCF-7, SGC7901 and human umbilical vein endothelial (HUVEC) cells.

Compounds		IC_50_ (μM) ^1^		
	A549	MCF-7	SGC7901	HUVEC
**1**	>50	2.65 ± 0.14	>50	>50
**2**	>50	>50	>50	ND ^3^
**3**	>50	>50	>50	ND
**4**	>50	>50	>50	ND
**5**	>50	>50	>50	ND
**6**	>50	>50	>50	ND
**7**	>50	>50	>50	ND
**8**	10.41 ± 1.02	19.44 ± 1.74	8.96 ± 0.99	ND
Taxol ^2^	0.047 ± 0.08	0.073 ± 0.11	0.069 ± 0.03	ND

^1^ IC_50_ values represent mean ± standard deviation of three individual observations; ^2^ Taxol was used as the positive control; ^3^ ND, not determined.
